# Modelling ponatinib resistance in tyrosine kinase inhibitor-naïve and dasatinib resistant *BCR-ABL1*+ cell lines

**DOI:** 10.18632/oncotarget.26187

**Published:** 2018-10-05

**Authors:** Liu Lu, Chung Hoow Kok, Verity Ann Saunders, Jueqiong Wang, Jennifer Anne McLean, Timothy Peter Hughes, Deborah Lee White

**Affiliations:** ^1^ South Australian Health and Medical Research Institute (SAHMRI), Cancer Theme, Adelaide, SA, Australia; ^2^ School of Medicine, Faculty of Health and Medical Sciences, University of Adelaide, Adelaide, SA, Australia; ^3^ Australian Centre for Blood Diseases, Monash University, Melbourne, VIC, Australia; ^4^ Discipline of Paediatrics, Faculty of Health and Medical Sciences, University of Adelaide, Adelaide, SA, Australia; ^5^ School of Biological Sciences, Faculty of Sciences, University of Adelaide, Adelaide, SA, Australia; ^6^ Department of Haematology, SA Pathology, Adelaide, SA, Australia

**Keywords:** chronic myeloid leukaemia, ponatinib resistance, Bcr-Abl+ cell lines, compound mutation, Axl

## Abstract

TKI resistance remains a major impediment to successful treatment of CML. In this study, we investigated the emerging modes of ponatinib resistance in TKI-naïve and dasatinib resistant *BCR-ABL1*+ cell lines. To investigate potential resistance mechanisms, ponatinib resistance was generated in *BCR-ABL1*+ cell-lines by long-term exposure to increasing concentrations of ponatinib. Two cell lines with prior dasatinib resistance demonstrated *BCR-ABL1* kinase domain (KD) mutation(s) upon exposure to ponatinib. In one of these cell lines the T315I mutation had emerged during dasatinib exposure. When further cultured with ponatinib, the T315I mutation level and *BCR-ABL1* mRNA expression level were increased. In the other cell line, compound mutations G250E/E255K developed with ponatinib exposure. In contrast, the ponatinib resistant cell lines that had no prior exposure to other TKIs (TKI-naïve) did not develop *BCR-ABL1* KD mutations. Rather, both of these cell lines demonstrated Bcr-Abl-independent resistance via Axl overexpression. Axl, a receptor tyrosine kinase, has previously been associated with imatinib and nilotinib resistance. Ponatinib sensitivity was restored following Axl inhibition or shRNA-mediated-knockdown of Axl, suggesting that Axl was the primary driver of resistance and a potential target for therapy in this setting.

## INTRODUCTION

Since its clinical introduction over a decade ago, imatinib has revolutionized the treatment of chronic phase-chronic myeloid leukaemia (CP-CML). However, approximately 30-40% of patients fail to respond optimally to imatinib [1]. The best-characterized mechanism of secondary imatinib resistance is the development of mutations within the *BCR-ABL1* kinase domain (KD). More than 100 mutations have been identified which either prevent or significantly disrupt the binding of imatinib to Bcr-Abl [2]. To overcome this, second generation tyrosine kinase inhibitors (TKIs) including nilotinib and dasatinib were developed. For many imatinib resistant patients, second generation TKIs are an effective salvage strategy. However, these TKIs are completely ineffective against the *BCR-ABL1* T315I mutation (commonly referred to as the gatekeeper mutation), which accounts for approximately 15-20% of clinically observed *BCR-ABL1* mutations [2, 3].

Ponatinib (Iclusig^®^, Ariad Pharmaceuticals, Cambridge, MA, USA) a third generation TKI, is a potent Bcr-Abl inhibitor approved in the USA and Europe for treatment of CML patients with resistance to other TKIs. The medium human peak and trough plasma levels of ponatinib when dosed at 45 mg once daily are 145 nM and 64 nM respectively [4]. Ponatinib was specifically designed on the basis of X-ray crystallographic analysis of the Abl kinase domain to target native and mutant isoforms of Bcr-Abl, including Bcr-Abl^T315I^. However, while ponatinib is the only available TKI to target Bcr-Abl^T315I^, the interaction of ponatinib with T315I mutant Bcr-Abl is weaker than its interaction with Bcr-Abl^p210^ [5, 6].

While ponatinib efficiently targets Bcr-Abl with a single KD mutation, multiple mutations in *BCR-ABL1* within the same clone, known as “compound mutations”, can occur and were found to confer ponatinib resistance [7]. Although only a minority of Philadelphia chromosome positive (Ph+) leukaemia patients harbour compound mutations, Zabriskie and colleagues [7] demonstrated that patients with 12 different compound mutations, including those that are T315I inclusive, are highly resistant to ponatinib and all other available TKIs.

In addition, a sub-optimal response to TKI therapy can be due to the development of other Bcr-Abl dependent mechanisms including reduced activity of the drug-influx transporter organic cation transporter 1 (OCT-1) [8–10], increased expression of drug-efflux ATP-binding cassette transporters, commonly *ABCB1* and *ABCG2* [8, 11–17], and/or *BCR-ABL1* over-expression [16, 18–20]. Moreover, patients who lose response to therapy without harbouring *BCR-ABL1* KD mutations are also observed in the clinic. Importantly, these patients may have adequate inhibition of Bcr-Abl activity [21], suggesting that Bcr-Abl independent mechanisms of resistance may drive the disease in these cases. Identified Bcr-Abl independent resistance mechanisms include the deregulation of PI3K signalling, Src family kinases, JAK-STAT signalling, and TAM (Tyro3, Axl, and Mer) family receptor tyrosine kinases, particularly Axl [22–27]. While the function of this kinase is yet to be determined, patients who are imatinib resistant were shown to have higher expression of *AXL* in a study by Dufies M *et al.* [22].

To investigate potential resistance mechanisms, ponatinib resistance was generated in this study by exposing *BCR-ABL1*+ cell lines to increasing concentrations of ponatinib. Cell lines were either TKI-naïve, or were previously treated with dasatinib to induce dasatinib resistance to mimic the clinical use of ponatinib in TKI-resistant/refractory CML & Ph+ALL patients, many of which had been on dasatinib with or without the T315I mutation. By characterising the resultant ponatinib resistant cell lines, we identified that in the setting of prior dasatinib exposure, Bcr-Abl dependent mechanisms cause ponatinib resistance. However, in the TKI-naïve setting, Bcr-Abl-independent modes of resistance dominate, and Axl presents as a key mediator of this resistance, suggesting Axl may be a potential therapeutic target in the cases of ponatinib resistance.

## RESULTS

### Four ponatinib resistant cell lines were generated

After long-term ponatinib culture, four ponatinib resistant cell lines were established (Table 1 and Supplementary Figure 1) and all were cross-resistant to imatinib, nilotinib and dasatinib. To investigate the ponatinib resistance, Bcr-Abl kinase sensitivity assays were performed (using phospho-CrkL as a surrogate measure of Bcr-Abl kinase activity) to determine the IC50 of ponatinib. Increased IC50 values indicated Bcr-Abl dependent ponatinib resistance in the two dasatinib pre-treated ponatinib resistant cell lines: K562 T315I-R (635 nM compared to control line 68 nM, p<0.001, n=3) and K562 DOX 55D-R cells (478 nM, compared to control line 51 nM, p<0.001, n=3), as higher concentrations of TKI were required to inhibit Bcr-Abl tyrosine kinase activity (Table 1, Supplementary Figure 2A-2B).

**Table 1:** Summary of the ponatinib-resistant cell lines, their parental controls and characteristised resistance mechanisms

Cell line name	Parental control	DAS pre-treated	Final ponatinib dose	Ponatinib pCkL IC50	TKI viability assay IC50	increased *BCR-ABL1* mRNA expression	Increased ABCB1 level	Increased ABCG2 level	KD mutation	Increased adherence	increased AXL mRNA expression	increased Axl protein level
**K562 T315I-R**	**K562 T315I**	✓	100 nM	635 nM (Control: 68 nM)	> 2 μM IM > 1μM NIL > 200 nM DAS	✓	X	X	✓ T315I	X	X	X
**K562 DOX 55D-R**	**K562 DOX 55D**	✓	200 nM	478 nM (Control: 51 nM)	> 2 μM IM > 1μM NIL > 200 nM DAS	X	X	X	✓ G250E+E255K	X	X	X
**K562-R**	**K562**	X	200 nM	6.6 nM (Control: 7.2 nM)	> 2 μM IM > 1μM NIL > 200 nM DAS	X	✓	X	X	✓	✓	✓
**K562 DOX-R**	**K562 DOX**	X	200 nM	11.3 nM (Control: 8.9 nM)	> 2 μM IM > 1μM NIL > 200 nM DAS	✓	X	X	X	✓	✓	✓
**KU812-R**	**KU812**	NA	Failed at 0.5 nM ponatinib	NA	NA	NA	NA	NA	NA	NA	NA	NA

Abbreviations: ✓ = yes; X = no. NA: not tested. IM: imatinib; NIL: nilotinib; DAS: dasatinib.

However, as demonstrated in (Supplementary Figure 2C), there was no significant difference (n=3) in the ponatinib IC50 of the K562-R cell line (6.6 nM) compared to the control cell line K562 (7.2 nM). In addition, the K562 DOX-R cells also demonstrated a similar ponatinib IC50 (11.3 nM, n=3) compared to the K562 DOX control (8.9 nM) (Supplementary Figure 2D). To confirm the resistance in these two TKI naïve ponatinib treated cell lines, viabilities were determined after 72 hours of ponatinib exposure. As shown in Supplementary Figure 3, both K562-R and K562 DOX-R cell lines were able to maintain high viabilities (>75%) in 200 nM ponatinib. These results suggested that Bcr-Abl independent mechanisms were likely involved in the development of ponatinib resistance in these two cell lines.

### Resistant cell lines demonstrated *BCR-ABL1* mRNA overexpression in the development of ponatinib resistance

**Figure 1:**
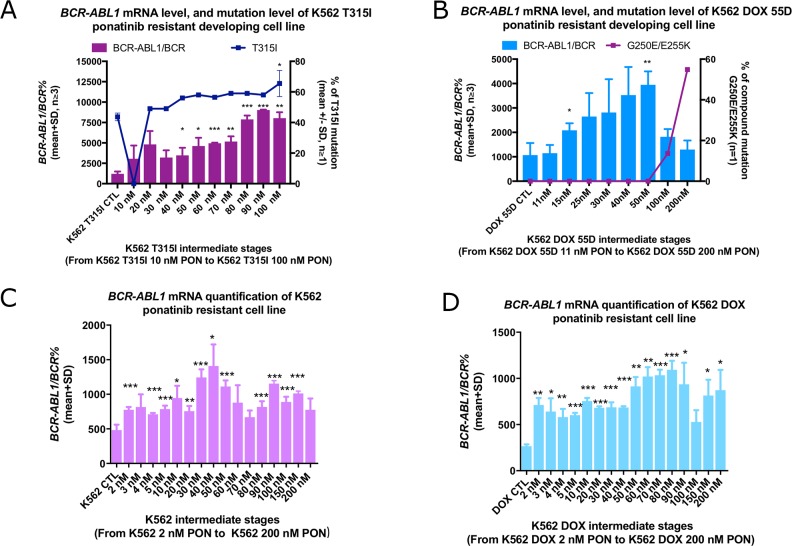
Increased T315I% was detected in the K562 T315I cell line during development of ponatinib resistance Reduction of *BCR-ABL1* mRNA overexpression coincided with the emergence of a compound mutation in the development of K562 DOX 55D-R ponatinib resistant cell line. Overexpression of *BCR-ABL1* mRNA level was observed in the intermediate cell lines, and the final **(A)** K562 T315I-R (100 nM) and **(D)** K562 DOX-R (200 nM) resistant cell lines, but not the **(B)** K562 DOX 55D-R (200 nM) and **(C)** K562-R (200 nM) resistant cell lines. (A) The percentage of T315I mutation was also increased in the K562 T315I-R (100 nM) cell line. (B) The reduction in the overexpression of *BCR-ABL1* in 100 nM intermediate followed the emergence of the compound mutation G250E/E255K. No mutation was detected in the (C) K562-R (200 nM) or (D) K562 DOX-R (200 nM) resistant cell lines. Error bars represent SD. mRNA expression represents the mean of at least three independent experiments performed in triplicate, ^*^p<0.05, ^**^p<0.01 and ^***^p<0.005 compared to the corresponding parental control lines (CTL).

Since overexpression of *BCR-ABL1* mRNA can cause resistance to first and second generation TKIs [23, 28, 29], RT-QPCR was performed to determine *BCR-ABL1* transcript number in the four ponatinib resistant cell lines. As expected, substantial increases in the expression level of *BCR-ABL1* mRNA were observed during the development of the K562 T315I-R and K562 DOX 55D-R cell lines. There was a significant increase in *BCR-ABL1* mRNA from 1206% (relative to %*BCR*) in naïve cells to 8027% in the K562 T315I-R resistant line (n=3, P<0.001) (Figure 1A). Moreover, *BCR-ABL1* RT-QPCR on the intermediate stages of resistance development (from 40 nM to 90 nM) revealed an increase in mRNA expression, peaking at 9034% in the 90 nM ponatinib intermediate, K562 T315I 90 nM PON (n=3, p<0.001) (Figure 1A). During the development of the K562 DOX 55D-R cell line, a step-wise increase in *BCR-ABL1* mRNA was also observed in the intermediate stages of resistance, from 1069% in the ponatinib naïve control cells and peaking at 3947% in the 50 nM ponatinib intermediate (n=3, P<0.001) (Figure 1B). This overexpression, however, then decreased to 1818% from the 100 nM intermediate stage onwards. The final K562 DOX 55D-R resistant cells (200 nM ponatinib) demonstrated a further reduction in *BCR-ABL1* mRNA expression (1299%), which was not significantly different to the ponatinib naïve control line K562 DOX 55D (1069%) (Figure 1B). This result suggests that the overexpression of *BCR-ABL1* mRNA may only facilitate early stage ponatinib resistance, and that other resistance mechanisms eventually predominate.

Notably, *BCR-ABL1* mRNA levels also increased during the development of K562-R and K562 DOX-R cell lines, however, the gene expression levels were significantly lower compared to the two ponatinib resistant cell lines which had developed prior resistance to dasatinib. As demonstrated in Figure 1C, the intermediate stages of the ponatinib resistance development in K562 exhibited fluctuations in the *BCR-ABL1* mRNA transcript level, from 487% in the K562 TKI-naïve control cells, peaking at 1408% in the 40 nM ponatinib culture (n=3, P<0.001). However, the K562-R final (200 nM ponatinib) resistant line did not exhibit a significantly increased *BCR-ABL1* mRNA level compared to its K562 control. This result suggested that the overexpression of *BCR-ABL1* mRNA may mediate early stages of ponatinib resistance, but it is not the major mechanism that caused resistance in this cell line. Conversely, the *BCR-ABL1* mRNA expression level in the K562 DOX-R ponatinib resistant line increased from 266% in the control line to 871% in the final 200 nM ponatinib culture (p=0.04, n=3) (Figure 1D). These results suggest that the overexpression of *BCR-ABL1* mRNA may mediate early stage ponatinib resistance, however, given the expression levels of *BCR-ABL1* were much lower in the prior TKI-naïve K562-R and K562 DOX-R cell lines than the other two ponatinib resistant lines, the over-expression is unlikely to be the only, or the dominant, resistance mechanism.

### No evidence of ABCG2 up-regulation in the ponatinib resistant lines, while ABCB1 was up-regulated in the K562-R ponatinib resistant cell line

Since overexpression of the efflux transporters ABCB1 and ABCG2 has been demonstrated to cause resistance to other TKIs [11, 16, 30, 31], the protein expression levels of these transporters were determined via flow cytometry in the ponatinib resistant cell lines. No surface expression of ABCB1 or ABCG2 was detected in the K562 T315I-R resistant or K562 T315I control cell lines (Supplementary Figure 4A and Supplementary Figure 5A). Flow cytometry did not detect ABCG2 expression in either of the K562 DOX 55D-R, K562-R or K562 DOX-R resistant cell lines nor in their corresponding parental controls (Supplementary Figure 4B-4D).

Notably, the K562 DOX 55D-R and the K562 DOX-R resistant cell lines demonstrated no changes in ABCB1 expression levels compared to their corresponding ponatinib naïve, ABCB1-overexpressing control lines (Supplementary Figure 5B, 5D). These results suggest that the ABCB1 overexpression was not required for the ponatinib resistance, hence ABCB1 overexpression is not a major cause of ponatinib resistance in the two cell lines.

Interestingly, while the control line K562 did not express cell surface ABCB1 (Supplementary Figure 5C), cell surface ABCB1 expression was observed in the K562-R ponatinib resistant culture, however the ponatinib IC50 in this cell line was similar to the control line. These data suggest that although ABCB1 might play a role in the development of resistance, it is unlikely that ABCB1 overexpression alters the intracellular concentration of ponatinib, and therefore this ABCB1 overexpression is not likely to mediate the observed ponatinib resistance by effluxing ponatinib.

### K562 T315I-R ponatinib resistant line demonstrated an increased *BCR-ABL1*^T315I^ mutation level

Ponatinib is a pan-Bcr-Abl inhibitor that effectively inhibits Bcr-Abl harbouring any of the identified single kinase domain mutations, including T315I. Previous results [28] revealed the control cell line K562 T315I to have 44% T315I *BCR-ABL1* KD gatekeeper mutation, causative of resistance to dasatinib, cross-resistant to imatinib and nilotinib, but sensitive to ponatinib. Conventional (Sanger) sequencing was performed on the intermediate cell lines generated during the development of ponatinib resistance and revealed a slow but steady increase in T315I% from 44% in the control line peaking at 66% (Figure 1A), with a comparatively reduced ponatinib sensitivity. Notably, during resistance development, the T315I mutation was undetectable in the 10 nM ponatinib intermediate (limit of detection is 10%), but was again present (at 49%) in the 20 nM ponatinib intermediate cell line (n=2).

### The percentage of T315I in cell lines corresponded to ponatinib sensitivity

**Figure 2:**
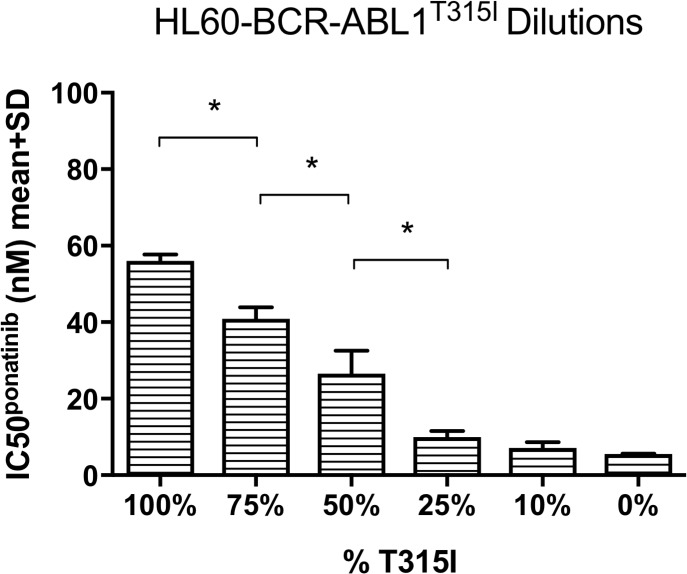
Ponatinib IC50 decreased in response to gradual reductions in the percentage of the T315I mutant Mean poantinib IC50 values were gradually reduced in the BCR-ABL1^T315I^ transduced HL60 cell line following dilution of the T315I with native BCR-ABL1^p210^ transduced HL60 cells. The reductions were significant in 75% (compared to 100%, p=0.004), 50% (compared to 75%, p=0.04) and 25% (compared to 50%, p=0.03) T315I dilutions (all n=3). ^*^p<0.05, data are mean + SD.

Ponatinib was designed to specifically target *BCR-ABL1*^T315I^ therefore it was necessary to confirm our observation that increased T315I mutation burden correlated with the development of ponatinib resistance. We assessed the effect of the T315I mutation burden on ponatinib sensitivity by conducting ponatinib IC50 analyses in an independent cell line - *BCR-ABL1* negative HL60 transduced with either *BCR-ABL1* p210 or *BCR-ABL1*^T315I^ (Figure 2 and Supplementary Figure 6A). HL60-*BCR-ABL1*^T315I^ cells were diluted in HL60-*BCR-ABL1* p210 cells to generate a T315I serial dilution. Mean ponatinib IC50 values reduced gradually from 56 nM in the 100% *BCR-ABL1*^T315I^ positive HL60 cell population, to 7 nM in the 10% T315I mutation dilution compared to 6 nM when no T315I was present in HL60-*BCR-ABL1* p210 (Figure 2). Statistically significant reductions were found in the cell populations with 75%, 50% and 25% T315I compared to the preceding dilutions (p<0.01, p=0.04 and p=0.03 respectively, all n=3). When dasatinib sensitivity was similarly assessed (Supplementary Figure 6B), incomplete kinase inhibition at even the highest dasatinib concentration rendered dasatinib IC50 values indeterminable for any percentage of T315I tested. These results suggest that if even a small proportion of a cell population was T315I-positive the mutant clone would result in overt resistance. In addition, although the sensitivity to ponatinib decreased with increasing T315I, both T315I-mutant cells and non-mutant cells were inhibited at pharmacologically relevant concentrations. Taken together, these dilution experiments confirm that the percentage of T315I in a cell population significantly impacts sensitivity to ponatinib treatment within clinically relevant ranges.

### K562 DOX 55D-R ponatinib resistant line harboured the compound mutations G250E and E255K

Conventional Sanger sequencing was unable to detect a BCR-ABL1 KD mutation in the K562-R and K562 DOX-R resistant cell lines. G250E (64%) and E255K (55%) were present in the K562 DOX 55D-R (200 nM) cell line, however neither the K562 DOX 55D ponatinib naïve control cell line, nor any of the intermediate cell lines, harboured a mutation detectable by Sanger sequencing (limit of detection is 10%).

Next, samples from the resistant line and the earlier intermediate stages were further analysed by next generation sequencing termed Single Molecule Consensus Sequencing (SMCS) [32] to investigate whether the mutations had emerged at an earlier time point (sensitivity <1%). As demonstrated in Figure 1B, G250E/E255K compound mutations were detected at 14% in the 100 nM intermediate K562 DOX 55D cell line. Interestingly, a single mutation E279K and a single mutation E255K were observed in 0.92% and 0.72% respectively in this 100 nM intermediate cell line. In the resistant line K562 DOX 55D-R (200 nM), the compound mutations G250E/E255K (54%) were also confirmed by SMCS (Figure 1B) and there was no other mutation detected.

### K562-R and K562 DOX-R ponatinib resistant lines demonstrated increased cell adhesion

**Figure 3:**
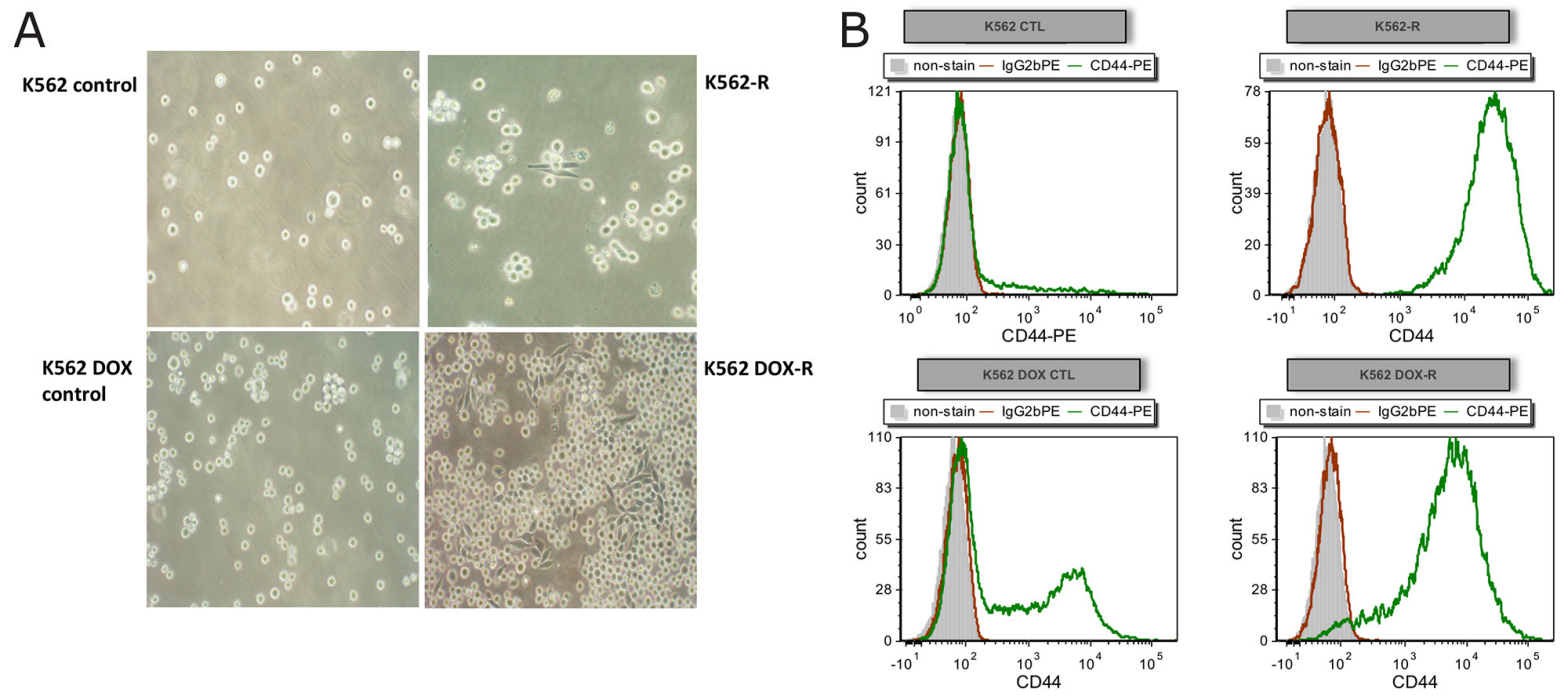
Resistant cell lines K562-R and K562 DOX-R demonstrate increased adhesion compared to their corresponding control lines **(A)** Adherent cells were observed in both resistant cell lines, but not in their respective control lines, by light microscopy with 20X magnification. **(B)** Cells from resistant and control cultures were analysed by flow cytometry for expression of the adhesion marker CD44 (CD44-PE green; unstained grey; IgG2b PE isotype control red).

Interestingly, the resistant cell lines K562-R and K562 DOX-R demonstrated increased adherence in culture as observed by morphology change, while the cells in the controls K562 and K562 DOX remained in suspension (Figure 3A). Due to this observation, flow cytometry was performed to assess the expression of the adhesion marker CD44 (E-selection, homing cell adhesion molecule or lymphocyte homing receptor) [33] in the two resistant cell lines and their respective controls. Increased adherence in K562 imatinib resistant cells (with CD44 staining positive) was reported in a previous publication [33]. As indicated in Figure 3B, the K562 control line was negative for CD44 while very small populations in the control line K562 DOX were positive for the adhesion marker. Conversely, the staining of CD44 was strongly positive in the two ponatinib resistant lines. This supported the morphology change observed in the resistant lines, and given that the increased adhesion was only observed in the two Bcr-Abl independent resistant cell lines, suggests that the adherence is likely to be associated with the Bcr-Abl independent mechanism of ponatinib resistance.

### K562-R and K562 DOX-R ponatinib resistant lines demonstrated Axl overexpression

**Figure 4:**
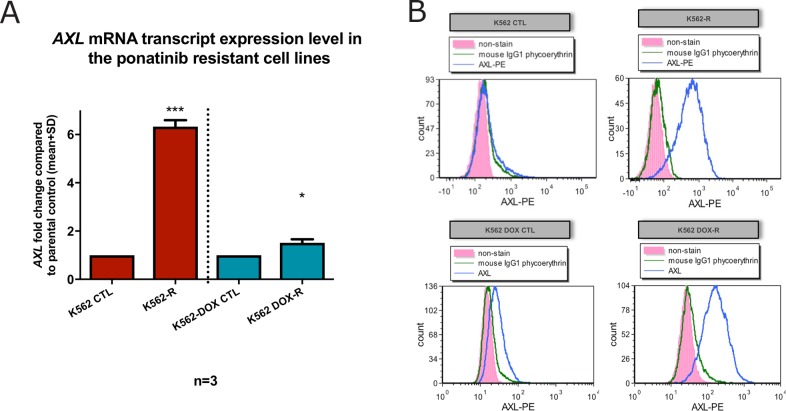
K562-R and K562 DOX-R cell lines demonstrated increased expression of the pro-adhesion protein Axl **(A)** *AXL* mRNA transcript was measured by RQ-PCR and the results were graphed as fold change compared to control lines. *GUSB* was used as control gene. Error bars represent SD. n=3 for all data. ^*^p<0.05 and ^***^p<0.005. **(B)** Flow cytometric analysis for AXL (anti-AXL-PE) was performed on resistant cell lines K562-R and K562 DOX-R and the respective control cell lines K562 and K562 DOX (unstained pink; IgG1 PE isotype control green; Axl PE antibody blue).

Increased adherence is a hallmark of Axl overexpression which has previously been associated with TKI resistance [22–27]. Therefore, *AXL* mRNA and Axl protein expression levels were assessed in the K562-R and K562 DOX-R cell lines. Figure 4A clearly demonstrates that *AXL* mRNA transcript level was significantly increased: a 6.3 fold increase in the K562-R line compared to the K562 control line (n=3, p<0.005); a 1.5 fold increase in the K562 DOX-R line compared to the K562 DOX control line (n=3, p=0.03).

Next, the Axl protein level was assessed in the two resistant cell lines by flow cytometry. As indicated in Figure 4B, the cell surface expression of Axl in the K562-R and K562 DOX-R resistant cell lines was dramatically increased compared to their corresponding control lines. Taken together, these data confirmed the overexpression of Axl in the two Bcr-Abl-independent resistant cell lines.

### Axl inhibition in the K562-R and K562 DOX-R ponatinib resistant lines restored sensitivity to ponatinib

**Figure 5:**
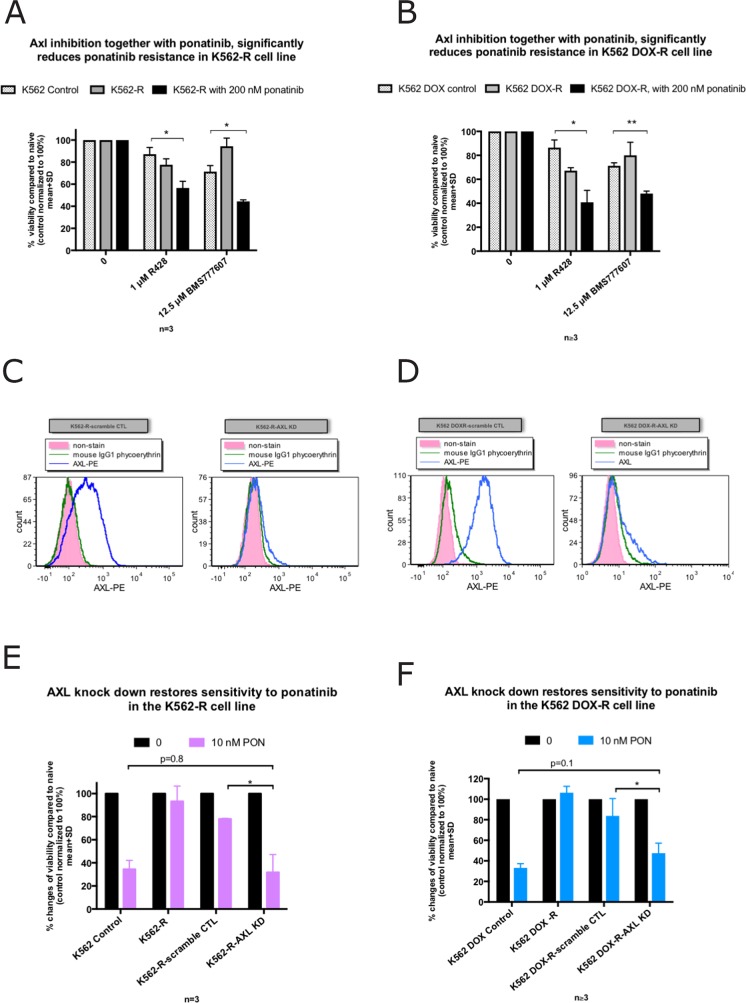
Inhibition of Axl restores ponatinib sensitivity in the K562-R and K562 DOX-R cell lines while *AXL* knockdown increases ponatinib sensitivity **(A, B)** Viability as assessed by Flow cytometry (AnnexinV-PE/7AAD double negative). The presence of the Axl inhibitor R428 (1 μM) or BMS777607 (12.5 μM) induced cell death significantly in (A) K562-R and (B) K562 DOX-R when co-treated with 200 nM ponatinib compared to their corresponding control lines. **(C, D)** Cell surface Axl expression was measured by flow cytometry in the AXL or scramble control shRNA transduced resistant cell lines. Compared to scramble control, *AXL* shRNA transduced cells demonstrated a reduction in Axl expression on the cell surface. **(E, F)** *AXL* knockdown in the K562 and K562 DOX ponatinib resistant cell lines demonstrated re-sensitisation to 10 nM ponatinib. Error bars represent SD, n≥3. ^*^p<0.05 and ^**^p<0.01.

Since Axl was overexpressed in the two resistant lines, we next determined if Axl inhibition might restore ponatinib sensitivity. A specific pharmacological Axl inhibitor, R428 was employed. R428 is 50 to 100-fold more specific in targeting Axl than the other TAM family members (Mer and Tyro3) [34–37]. Additionally, MET-branded inhibitor BMS-777607, which is 3 times more potent against Axl than MET, was also employed. Figure 5A-5B demonstrated that in the presence of 1 μM R428 or 12.5 μM BMS777607 (both concentration chosen based on previous publications) [38–40], and 200 nM ponatinib (the culture concentration of ponatinib), the viability of K562-R (n=3, p=0.02 and p=0.03 respectively) and K562 DOX-R (n=4, p=0.02 and p<0.01 respectively) resistant lines was significantly reduced compared to their corresponding control lines.

Taken together, these results indicate that Axl inhibition restored ponatinib sensitivity in the two Bcr-Abl independent resistant cell lines, suggesting that Axl is critical in mediating Bcr-Abl independent ponatinib resistance in these cell lines.

### *AXL* knock-down in the K562-R and K562 DOX-R ponatinib resistant lines restored sensitivity to ponatinib

To further confirm that Axl plays a role in the Bcr-Abl independent ponatinib resistance, *AXL* was knocked-down in the K562-R, and K562 DOX-R resistant cell lines by Lentiviral shRNA (with a GFP tag). Stable *AXL* knock-down K562-R-AXL KD, and K562 DOX-R-AXL KD cell lines were generated, as well as the scramble control lines K562-R-scramble CTL, and K562 DOX-R-scramble CTL. The positive expression of GFP in the *AXL* knock-down and scramble cell lines indicated the success of the transduction (Supplementary Figure 7A, 7B), which was further confirmed by the dramatic reductions in Axl expression in the knock-down cell lines (Figure 5C, 5D).

Next, the successfully transduced cell lines were exposed to ponatinib to determine whether *AXL* knock-down reduced ponatinib resistance. As demonstrated in Figure 5E), the K562-R-AXL KD cell line exhibited a dramatic decrease (68% reduction) in viability after incubating with 10 nM ponatinib for 72 hours. This reduction was significantly higher (n=3, p=0.03) compared to the reduction in the control line treated with scrambled shRNA (22% reduction). In addition, the level of this reduction was similar to the K562 parental ponatinib naïve control (n=3), suggesting that *AXL* knock-down restores ponatinib sensitivity in the K562-R resistant cell line to the parental control level.

The *AXL* knock-down in the K562 DOX-R resistant cell line also demonstrated a significant decrease in viability (53% reduction) compared to the corresponding control line treated with scrambled shRNA (16% reduction) (n=3, p=0.02) (Figure 5F) after incubating with 10 nM ponatinib for 72 hours. Again, the extent of cell death in the presence of 10 nM ponatinib following *AXL* knock-down was not significantly different to the K562 DOX parental control (n=3). This result suggested that *AXL* knock-down is responsible for restoring ponatinib sensitivity in the K562 DOX-R resistant cell line, to a level that was comparable to that observed in the ponatinib naïve parental control.

## DISCUSSION

*In vitro* cell line models have been used extensively to characterise resistance to the first and second generation TKIs in CML: our group and others have previously generated imatinib, nilotinib and dasatinib resistance in human and/or murine *BCR-ABL1*+ cell lines by culturing with gradually increasing concentrations of TKIs in a step-wise manner [16, 19, 28, 29]. TKI resistance mechanisms generated by this method were found to be similar to those observed in the clinic [16, 19, 28, 29]. Hence the same method was employed in the current study to investigate ponatinib resistance.

Five *BCR-ABL1*+ cell lines were chosen as *in vitro* models for ponatinib resistance development, with resistance successfully generated in 4 of the 5 cell lines (Table 1). Variable ponatinib responses were observed in different cell lines but resistance to ponatinib was shared by all of the resistant cell lines despite the different resistant mechanisms. Of note, after three times attempted, ponatinib resistance was not achieved by using KU812 cell line model. KU812 cells contained less copies of *BCR-ABL1* compared to the K562 and K562 variants, hence it is much sensitive to ponatinib.

Ponatinib resistance was generated in two dasatinib pre-treated cell lines, K562 T315I and K562 DOX 55D, and Bcr-Abl dependent mechanisms were identified to confer resistance. The K562 T315I-R ponatinib resistant line demonstrated an increased in the percentage of the T315I mutation and an increase in *BCR-ABL1* mRNA transcript level. The K562 DOX 55D-R resistant line also demonstrated Bcr-Abl dependent resistance, with the emergence of the compound mutations G250E/E255K. This result is in line with a previous study demonstrating that sequential ponatinib-inclusive TKI treatments could result in the development of compound mutations [7]; and while ponatinib efficiently targets all of the identified single KD mutations, some compound mutations can induce ponatinib resistance [7]. Interestingly, cases of G250E and E255K mutations appearing in imatinib or ponatinib treated patients were reported recently [41, 42], although, whether these two mutations were compounds was unclear. Hence, while the G250E/E255K compound mutations have not previously been reported, this study suggests that these compound mutations are likely to develop and confer resistance to ponatinib and other TKIs. Furthermore, a previous study showed that the P-loop mutation E255K in Bcr-Abl was close to the binding site of ponatinib and could affect the ponatinib efficacy. However, another P-loop mutation by itself, G250E, did not directly affect ponatinib binding [43]. While the structural interaction of ponatinib with Bcr-Abl harbouring both of these mutations remains unclear, the insensitivity of the K562 DOX 55D-R cell line to ponatinib confirms that an E255K/G250E compound mutations result in ponatinib resistance *in vitro*.

The K562-R and K562 DOX-R cell line models indicate that increased survival signalling through alternative kinase driven pathways, regardless of Bcr-Abl activity, play a role in the development of ponatinib resistance in the TKI naïve setting, as evidenced by the activation of Axl in this study. Previous publication indicates that *AXL* mRNA overexpression has been associated with imatinib and nilotinib resistant CML and therefore this gene alteration could be a potential biomarker to predict TKI treatment failure [22]. Here, both *AXL* mRNA and Axl protein overexpression was observed in the resistant cell lines. With Axl inhibition via small molecule inhibitors, ponatinib effectively induced apoptosis in the cell lines. In addition, the knock-down of *AXL* completely restored ponatinib sensitivity. These results indicated that Axl overexpression is critical in mediating survival and resistance. Of note, *AXL* knock-down is more effectively induced ponatinib cell death compared to the use of inhibitors. This may be due to shRNA is more specific in targeting Axl compared to the Axl inhibitors. This finding is in line with a recent publication that suggested that inhibition of Axl re-sensitised the ponatinib resistant cell lines and patient cells [44].

Axl inhibition re-sensitised the two resistant cell lines to ponatinib suggesting that combination therapy may be warranted in the Bcr-Abl independent ponatinib resistant setting. As the ATP-competitive Axl inhibitor R428 is already in Phase I clinical trial in acute myeloid leukaemia and Phase II trial in lung cancer, this provides a pathway for clinical studies in CML. Moreover, measurement of Axl expression level by flow cyotometry could potentially be used to risk-stratify patients who receive ponatinib therapy.

In conclusion, the work presented here demonstrates that ponatinib resistance can be generated *in vitro* and is therefore likely to be clinically relevant, as it is for all the first and second generation TKIs. This is the first study to present evidence that an increased proportion of T315I decreases sensitivity to ponatinib, and the emergence of compound mutations G250E/E255K result in ponatinib resistance. More importantly, in the context of recent clinical studies where ponatinib has been used in the early stages of CML therapy, Axl overexpression was identified to confer Bcr-Abl independent resistance in the TKI-naïve setting. Accordingly, inhibition of Axl reverses resistance to ponatinib. From a clinical perspective, examination of Axl expression level in patient leukaemic cells may provide a predictor for their likely response to ponatinib therapy. Furthermore, determination of Axl protein expression level in patients insensitive to ponatinib therapy could be useful in designing rational combination therapeutic interventions for these patients.

## MATERIALS AND METHODS

### Cell lines

The human *BCR-ABL1* negative cell line HEK 293T and HL60, and *BCR-ABL1* positive cell lines K562 and KU812 were purchased from The American Type Tissue Culture Collection (Manassas, USA). The ABCB1-overexpressing K562 variant, K562 DOX, was kindly provided by Prof. Leonie Ashman [45]. Two dasatinib resistant cell lines previously generated by Tang *et al.* [28] were used in this study : K562 T315I (named K562 200nM DAS in the original paper) and K562 DOX 55D which had prior exposure to 200 nM and 55 nM dasatinib respectively (Supplementary Figure 1). The T315I mutation was present in the K562 T315I cell line while no mutation was detected in the K562 DOX 55D cell line.

To generate ponatinib resistance, all *BCR-ABL1*+ cell lines (Table 1 and Supplementary Figure 1) were cultured with increasing concentrations of ponatinib using methods previously described [16, 28]. Parental, serially passaged ponatinib-naïve K562, K562-DOX, K562-DOX 55D and K562 T315I cell lines and 0.1% Di-methyl Sulfoxide (DMSO; Merck, Darmstadt, Germany) vehicle controls were maintained in parallel.

#### IC50 analysis

IC50 experiments for imatinib, nilotinib, dasatinib and ponatinib were determined by measuring the inhibition of the Bcr-Abl substrate CrkL by using the method as outlined by White *et al.* [46]. In brief, 2 × 10^5^ cells were incubated in the presence of TKI at 37°C (5% CO_2_) for 2 hours. After incubation, cells were washed and lysed in Laemmli buffer [27]. Proteins were resolved using sodium dodecyl sulfate polyacrylamide gel electrophoresis (SDS-PAGE). Western blots were performed by using anti-CrkL antibody C20 (Santa Cruz Biotechnology). The percentage of p-CrkL to total CrkL was determined, and the concentration of TKI required to reduce pCrkL by 50% was recorded as the IC50 value.

#### Tyrosine kinase inhibitors and Axl inhibitors

Ponatinib (Selleckchem, Houston, USA), dasatinib (Symansis, Shanghai, China) and nilotinib (Symansis) were dissolved in DMSO (10mM). Imatinib (Symansis) was dissolved in water (10mM). Serial dilutions of all drugs were made immediately prior to use.

Axl inhibitors R428 and BMS-777607 (both from Selleckchem, Houston, USA) were dissolved in DMSO at 10 mM. Serial dilutions were made in DMSO immediately prior to use.

#### BCR-ABL1 mRNA quantification and BCR-ABL1 kinase domain sequencing

*BCR-ABL1* quantification was performed as previously described [28, 47]. In brief, RNA was extracted from cells stored in Trizol solution (Invitrogen Australia, Australia). cDNA was synthesized using Superscript II (Invitrogen) and was used as a template in a quantitative PCR (RQ-PCR) reaction using the ABI Prism 7500 sequencing detection system (Applied Biosystems, Australia). *BCR-ABL1* transcript numbers were reported as *BCR-ABL1/BCR%*.

Sanger sequencing was performed as previously described [31, 47]. Briefly, RNA was extracted from 2 × 10^6^ cells, and cDNA synthesized as a template to amplify the KD of *BCR-ABL* in a long PCR reaction using the Expand Long Template PCR System (Roche, Australia). Products were purified using ExoSAP-IT (GE Healthcare, Sweden) and sent to Molecular Pathology (SA Pathology) for analysis.

Next generation sequencing (NGS) termed Single Molecule Consensus Sequencing (SMCS) was performed using previous method and kindly processed by Dr. Wendy Parker [32].

#### Immunophenotyping by Flow Cytometry: measurement of protein cell surface expression

5 × 10^5^ cells were incubated with Hanks Balanced Salt Solution (HBSS) (JRH Biosciences, Australia) supplemented with 10mM Hepes (Sigma-Aldrich) and with corresponding isotype controls: IgG2aPE/IgG2bPE (Dako, Australia), IgG1PE (BD Biosciences, Australia); or PE-conjugated anti-hBCRP1 (ABCG2-PE) (R&D Systems, Australia), PE-conjugated anti-CD243 (ABCB1-PE) (Beckman Coulter, Australia), PE-conjugated anti-hAxl (R&D Systems, Australia) or PE-conjugated anti-hCD44 (BD Biosciences, Australia) antibodies for 40 minutes on ice. Following HBSS wash, cells were resuspended in fixative and analyzed by flow cytometry (BD LSRFortessa X20 Analyser, Australia). Results were analyzed by FCS Express 4 Research Flow Edition software (De Novo, US).

### *AXL* transcript quantitation

*AXL* transcript levels were measured by RQ-PCR. *GUSB* was used as control gene and the expression level of *AXL* was calculated as a ratio of *GUSB* transcript levels. The primers used for *AXL* amplification were as follows:

*AXL* Forward primer: 5′ TGC ATG AAG GAA TTT GAC CA 3′

*AXL* Reverse primer: 5′ TCG TTC AGA ACC CTG GAA AC 3′

Samples were amplified in triplicate in the Rotor-Gene 3000 PCR cycler machine (Corbett Research, Cambridgeshire, UK) with the following conditions: 50°C for 2 min, 95°C for 10 min, 45 cycles of 95°C for 15 seconds and 60°C for 1 min.

### Viability assay

Cells were removed from drug-containing culture medium, washed and re-cultured in fresh media (with testing drug) for 72 hours. Cells then were stained with 7-Amino-actinomycin D (7AAD) and Annexin V-PE. Flow cytometry (BD LSRFortessaTM X-20 with high-throughput platform) was performed to determine cell viability: cells stained positive for Annexin V-PE and 7AAD were considered to be apoptotic or dead respectively. The data were analysed using FCS Express software (DeNovo Software, Los Angeles, California, USA).

### Lentiviral transfection and transduction

HEK 293T cells were transfected by lentiviral shRNA plasmid pGFP-C-shLenti comprised of either scramble control vector (sequence as below) or AXL knock-down (sequence as below) with Green Fluorescense Protein (GFP; Origene, MD, USA) according to the manufacturer's instructions. The GFP level and Axl expression level were measured after fluorescence-activated cell sorting (FACS) and before each experiment to ensure purity.

Scramble control sequence:

5′ GCA CTA CCA GAG CTA ACT CAG ATA GTA CT 3′

*AXL* shRNA sequences:

5′ GAA CAG GAT GAC TGG ATA GTG GTC AGC CA 3′

#### Statistics

Comparison between two groups was performed using unpaired 2-tailed Student's *t*-test or Kruskal-Wallis analysis of variance to determine statistical differences. Correlation between 2 groups was compared using Pearson's correlation statistical analysis. The analysis was performed using GraphPad Prism 5 (GraphPad Software, La Jolla, CA). Statistically significant differences were defined as p<0.05.

## SUPPLEMENTARY MATERIALS FIGURES


