# Supplementary material for: Modelling ponatinib resistance in tyrosine kinase inhibitor-naïve and dasatinib resistant BCR-ABL1+ cell lines
Source: Oncotarget. 2018 Oct 5;9(78):34735–47. doi: 10.18632/oncotarget.26187 (PMC6205183; doi:10.18632/oncotarget.26187)
Supplement: Supplementary file 1 [file oncotarget-09-34735-s001.pdf]

Modelling ponatinib resistance in tyrosine kinase inhibitor-naïve and dasatinib resistant *BCR-ABL1*+ cell lines

SUPPLEMENTARY MATERIALS

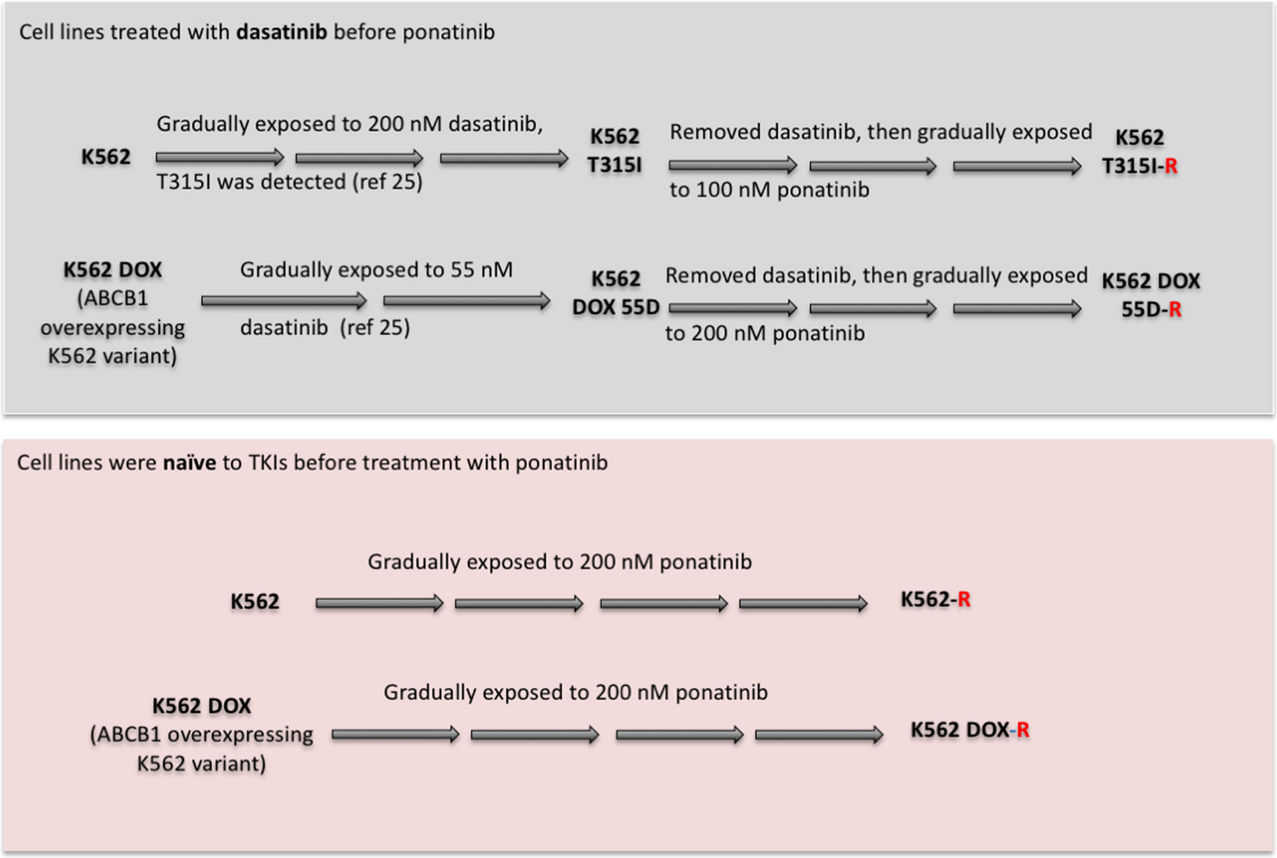

Supplementary Figure 1: Schematic summary of ponatinib resistance generation in dasatinib treated or TKI naïve cell lines.

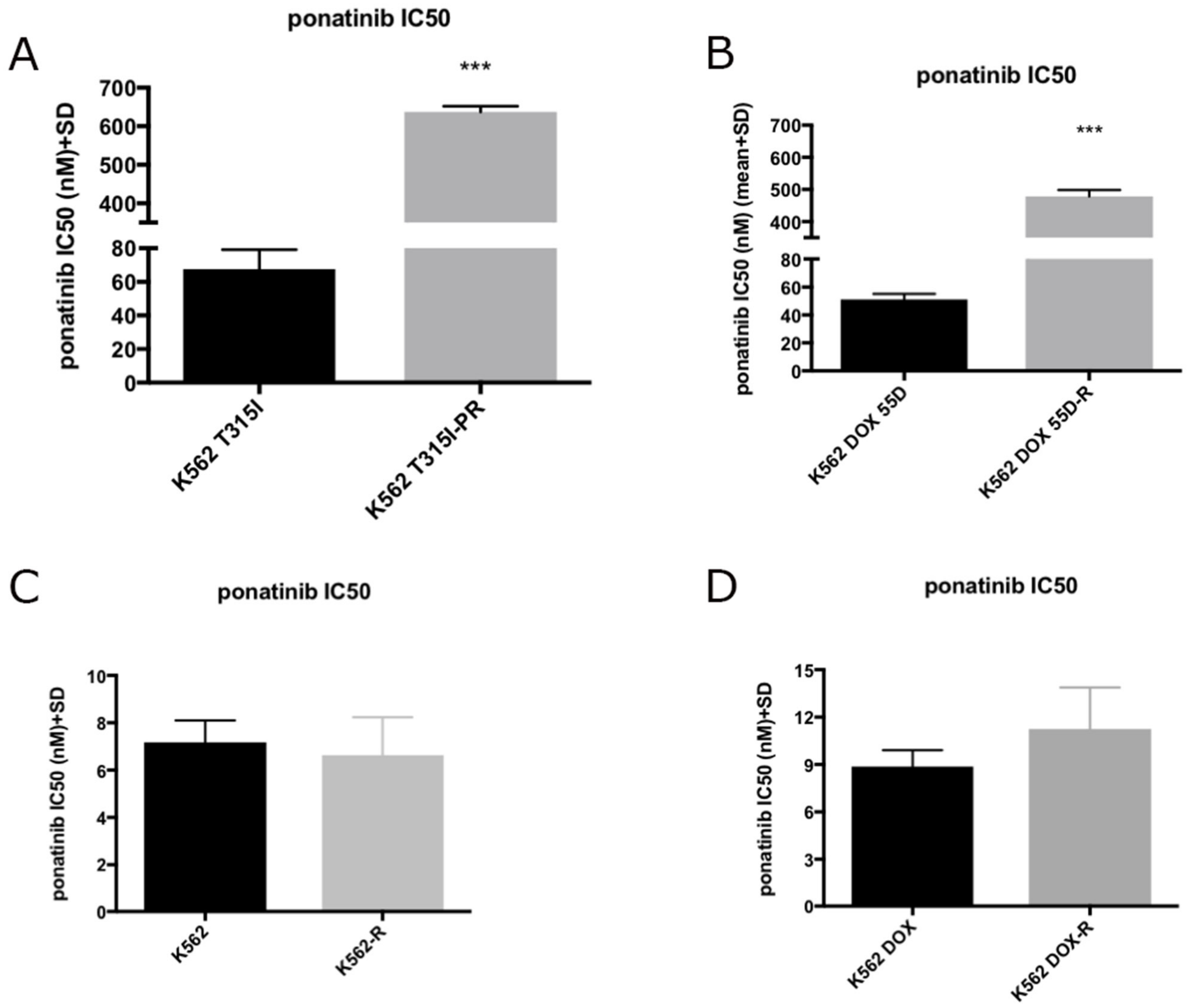

Supplementary Figure 2: ponatinib IC50 values for K562 T315I-R and K562 DOX 55D-R were significantly increased compared to their corresponding control lines. Cells were incubated for 2 hours with serial concentrations of ponatinib and then lysed to perform p-CrkL western blotting. **(A, B)** Increased ponatinib IC50s were observed in the K562 T315I-R and K562 DOX-R cell lines compared to their corresponding control lines K562 T315I and K562 DOX 55D. **(C, D)** Similar ponatinib IC50s were observed in the K562-R and K562 DOX-R cell lines compared to their corresponding control lines K562 and K562 DOX. Error bars represent SD, $n \geq 3$, $^{*}p<0.05$, $^{**}p<0.01$ $^{***}p<0.005$.

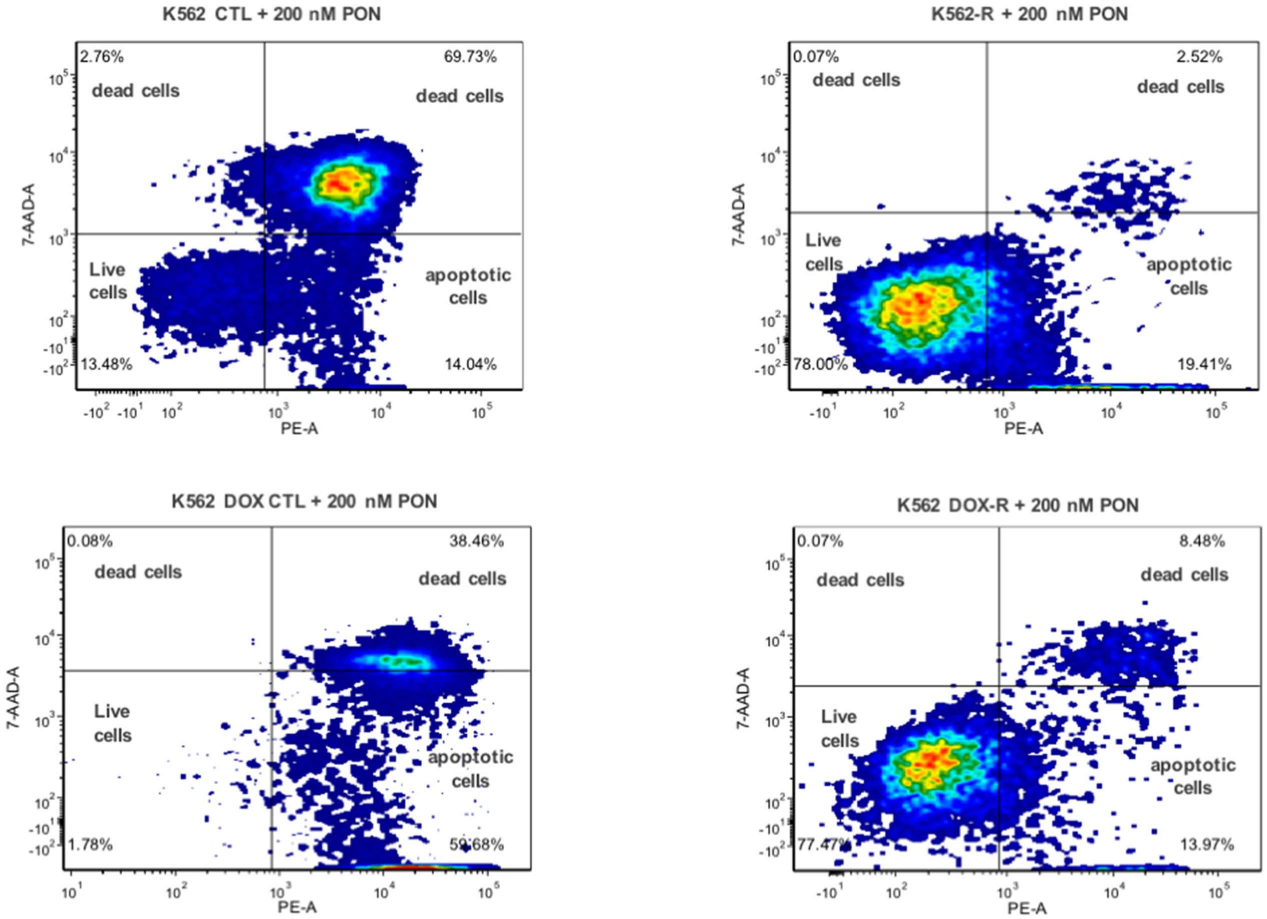

Supplementary Figure 3: K562-R and K562 DOX-R cells are resistant to high level of ponatinib. Cells from the resistant and their control cell lines were cultured with 200 nM ponatinib concentrations for three days and stained with 7-AAD and annexin V for viability detection. Both K562 and K562 DOX control cell lines demonstrated sensitivity ponatinib while the two resistant cell line demonstrated viability over 75% after ponatinib culturing.

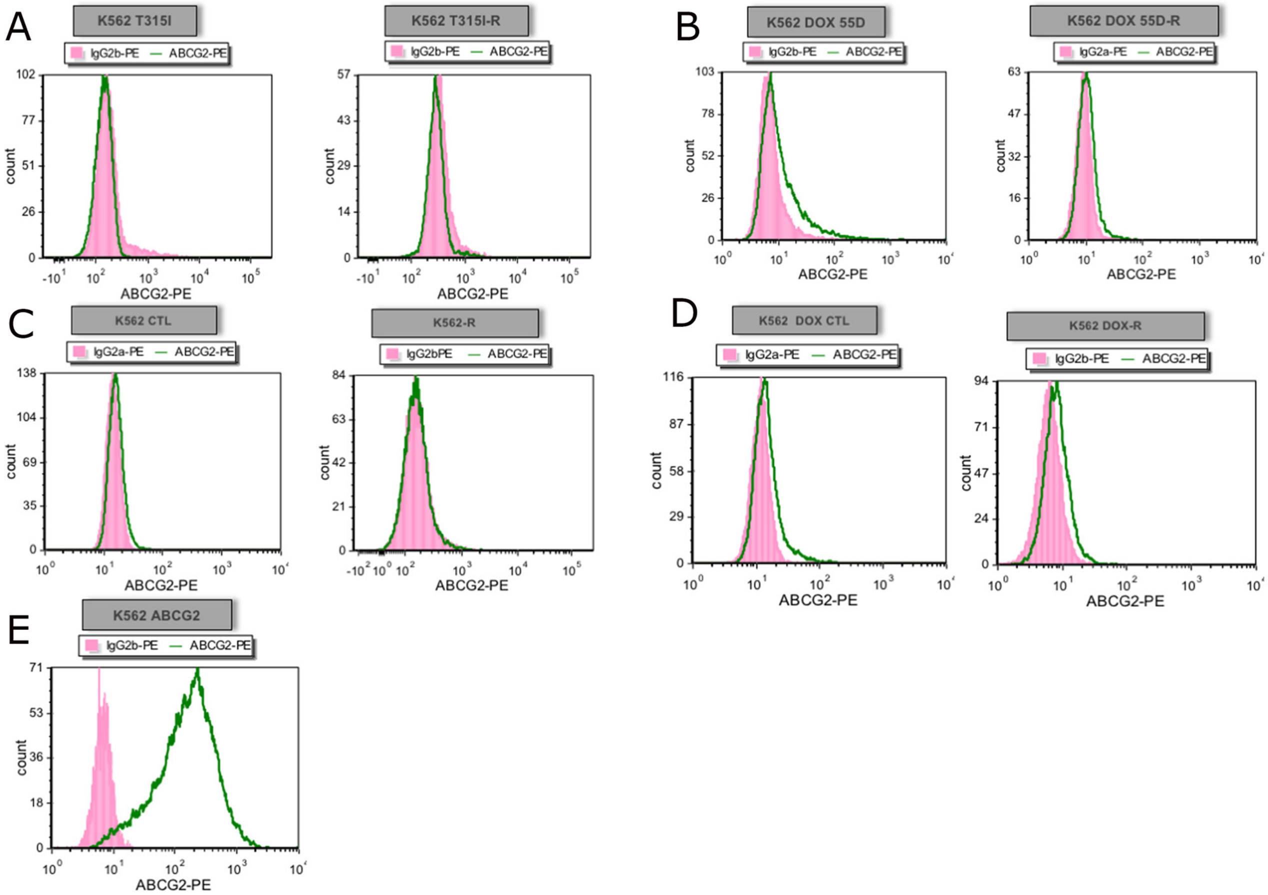

Supplementary Figure 4: cell surface ABCG2 expression was undetectable in the ponatinib resistant cell line. Cells from the resistant and their control cell lines were collected and stained with either IgG2b PE isotype control antibody (pink) or the corresponding ABCG2 PE antibody (green). **(A, B, C, D)** Flow cytometry staining ABCG2 in the control (left) and resistant cells (right). ABCG2 staining were negative among of the tested resistant cell lines. **(E)** K562 ABCG2 cell line was used as a positive control for ABCG2 staining.

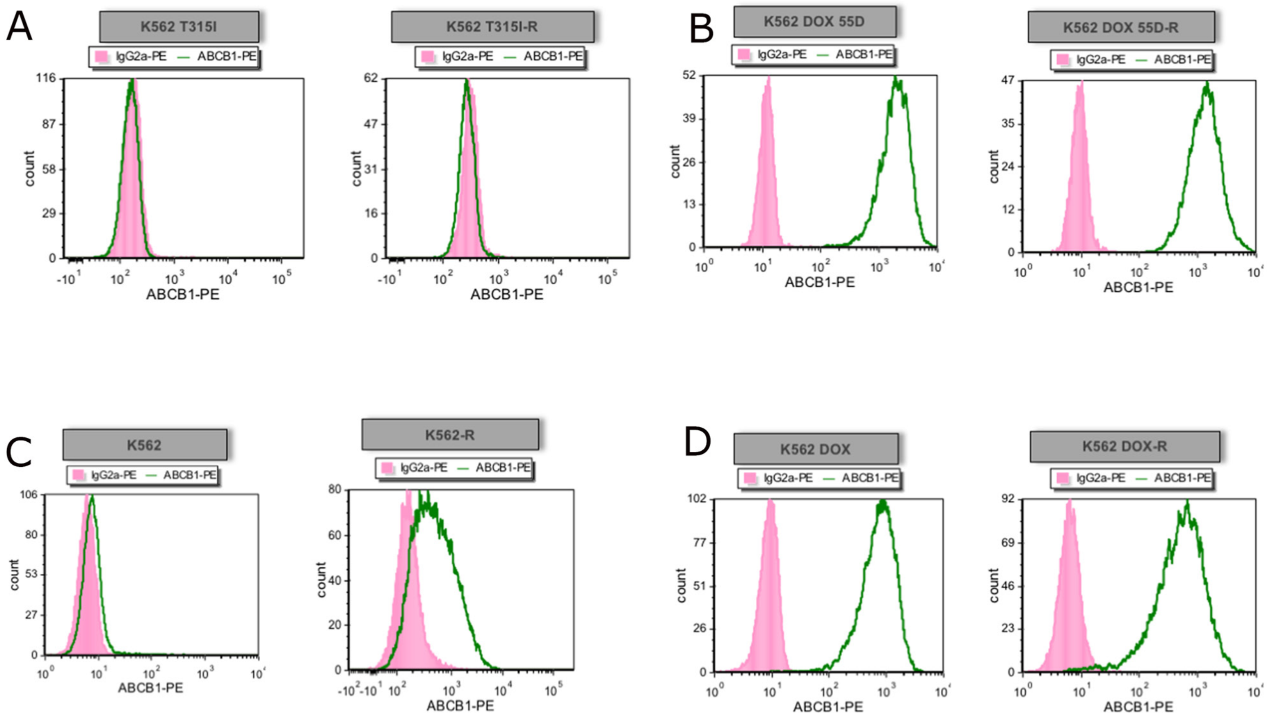

Supplementary Figure 5: ABCB1 overexpression was detected in the K562-R cell line. Cells from the resistant and their control cell lines were collected and stained with either IgG2a PE isotype control antibody (pink) or the corresponding ABCB1 PE antibody (green). Flow cytometry staining ABCB1 in the control (left) and resistant cells (right). **(A)** K562 T315I-R, **(B)** K562 DOX 55D-R and **(D)** K562 DOX-R demonstrated similar ABCB1 expression level compared to their corresponding controls, while **(C)** K562-R is the only resistant cell line demonstrated cell surface ABCB1 overexpression compared to the K562 control cell line.

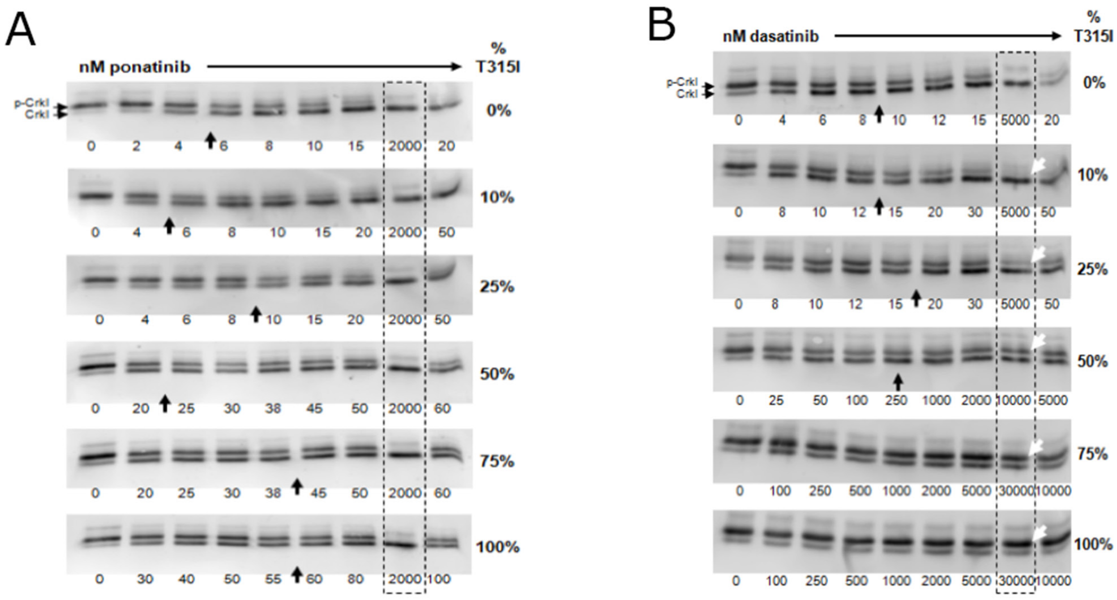

Supplementary Figure 6: Western blotting of ponatinib IC50 decreased in response to gradual reductions in the percentage of the T315I mutant. **(A)** Example of the ponatinib IC50 western blots of the BCR-ABL1^{T315I} transduced HL60 cell line **(B)** Graphical representation of dasatinib IC50 assays of the BCR-ABL1^{T315I} transduced HL60 cell line. Dasatinib was unable to prevent phosphorylation of CrkL at any concentration for either cell line. n=1.

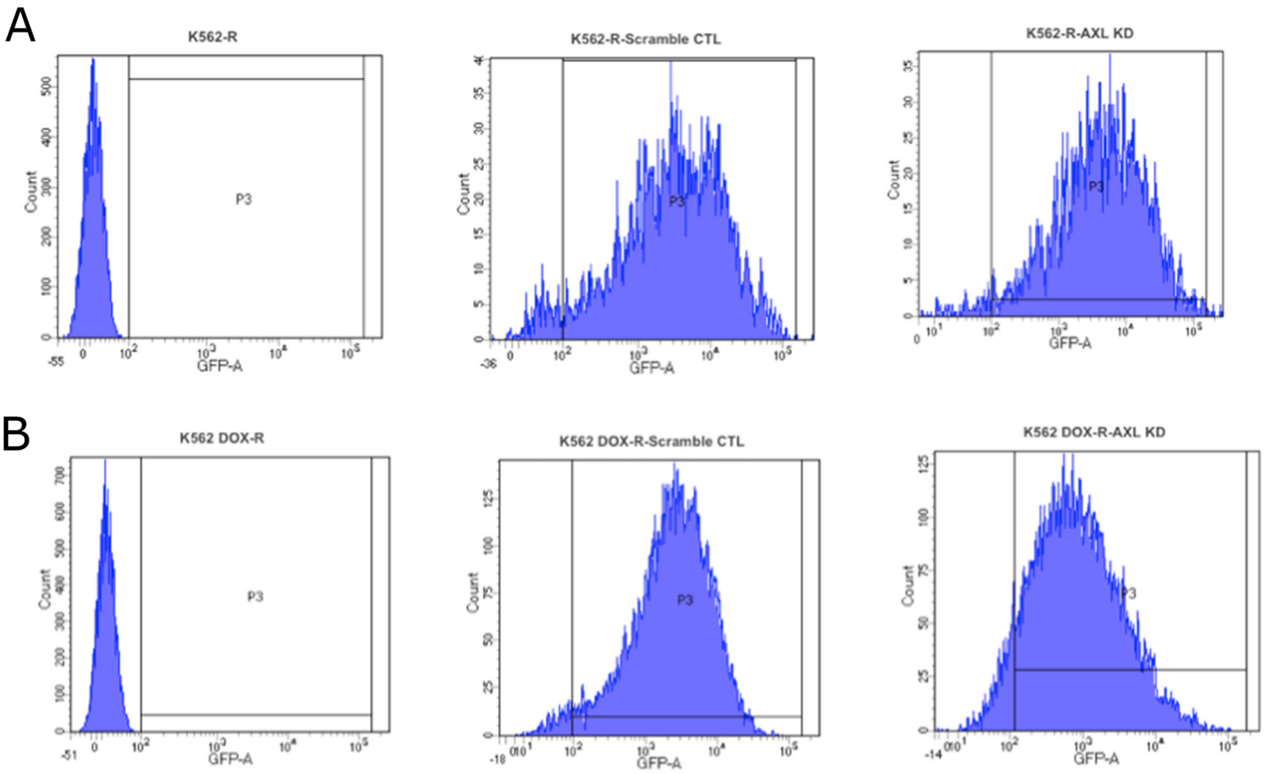

Supplementary Figure 7: GFP expression was detected by flow cytometry following transduction of the resistant cell lines with *AXL* or scramble control shRNA. The GFP expression in both **(A)** K562-R and **(B)** K562 DOX-R transduced cells was above 87% and in the experimental control (non-transduced) cells was <1%.
